# Community treatment orders in a Swedish county - applied as intended?

**DOI:** 10.1186/1756-0500-7-879

**Published:** 2014-12-06

**Authors:** Lars Kjellin, Veikko Pelto-Piri

**Affiliations:** Psychiatric Research Centre, Örebro County Council, and School of Health and Medical Sciences, Örebro University, Örebro, Sweden

**Keywords:** Psychiatry, Community treatment orders, Outpatient commitment, Compulsory community treatment, Mental health law reform

## Abstract

**Background:**

Community treatment orders (CTOs) were legally implemented in psychiatry in Sweden in 2008, both in general psychiatry and in forensic psychiatric care. A main aim with the reform was to replace long leaves from compulsory psychiatric inpatient care with CTOs. The aims of the present study were to examine the use of compulsory psychiatric care before and after the reform and if this intention of the law reform was fulfilled.

**Methods:**

The study was based on register data from the computerized patient administrative system of Örebro County Council. Two periods of time, two years before (I) and two years after (II) the legal change, were compared. The Swedish civic registration number was used to connect unique individuals to continuous treatment episodes comprising different forms of legal status and to identify individuals treated during both time periods.

**Results:**

The number of involuntarily admitted patients was 524 in period I and 514 in period II. CTOs were in period II used on relatively more patients in forensic psychiatric care than in general psychiatry. In all, there was a 9% decrease from period I to period II in hospital days of compulsory psychiatric care, while days on leave decreased with 60%. The number of days on leave plus days under CTOs was 26% higher in period II than the number of days on leave in period I. Among patients treated in both periods, this increase was 43%. The total number of days under any form of compulsory care (in hospital, on leave, and under CTOs) increased with five percent. Patients with the longest leaves before the reform had more days on CTOs after the reform than other patients.

**Conclusions:**

The results indicate that the main intention of the legislator with introducing CTOs was fulfilled in the first two years after the reform in the studied county. At the same time the use of coercive psychiatric care outside hospital, and to some extent the total use of coercive in- and outpatient psychiatric care, increased. Adding an additional legal coercive instrument in psychiatry may increase the total use of coercion.

## Background

During the last 20 – 25 years many countries and states have implemented legal regulations of coercive psychiatric care in the community [1], often labelled ‘Community Treatment Orders’ (CTOs). This form of treatment, in the literature also named for example compulsory community (mental health) treatment or care, (involuntary) outpatient commitment/treatment, supervised community treatment, or involuntary community treatment, implies that the patient is allowed to live in the community but is required to comply with certain conditions, such as taking his or her medication.

The use of coercion in outpatient settings is controversial [1–3], and the knowledge of how CTOs are applied and of their effects is limited [4–6]. There are studies reporting positive effects of CTOs, for example on time to hospital readmission [7] and on repeated hospitalizations [8]. In a Cochrane review, based on two randomized studies, it was however found that compulsory community treatment has no significant effect on service use, social functioning, or quality of life compared with standard care, but that people receiving community care were less likely to be victims of crime [9]. In a more recent RCT, the OCTET study, it was found that compulsory supervision outside hospital does not reduce the rate of readmission of psychotic patients [10]. Critics have however argued that there is a number of problems with the OCTET study, one of which being that it could not include the most important target group of patients, namely those who refused to participate in the research [11]. Research so far gives no clear picture of whether the total use of coercion in psychiatry will increase or decrease by implementing CTOs as a legal opportunity, but an increase over time in the use of CTOs has been reported from different countries [12, 13].

CTOs were introduced by a legal reform in Sweden which came into force in 2008, both in general psychiatry and in forensic psychiatric care. The law reform was mainly motivated by efforts to provide possibilities for a better and individually applied treatment and care [14]. In the government’s proposal for the legal change it was stated that many of the involuntarily treated patients in both general and forensic psychiatry were on temporary leaves in the community for long periods of time [15]. Leaves from compulsory care were thus used in a way that was not intended by the legislator, and a main aim with the reform was consequently to replace long leaves from compulsory psychiatric inpatient care with CTOs. In evaluations of the reform the Swedish National Board of Health and Welfare (NBHW) found differences in the way CTOs were implemented in general and forensic psychiatry [16, 17], but these evaluations did not study the impact on the amount of use of compulsory psychiatric care.

The overall objective of the present study was to examine the use of compulsory psychiatric care before and after the reform and if the main intention of the law reform was fulfilled. The more specific aims were:

- to describe the distributions on sex and diagnoses among involuntarily admitted inpatients, patients with CTOs and patients on leave before and after the reform,
- to describe the amount (patients, occasions, and days) of CTOs and leaves from compulsory psychiatric care after the reform compared to the amount of leaves before the legal change came into force,
- to analyse if the total amount of in- and outpatient compulsory psychiatric care increased or decreased after the reform,
- to analyse if the use of compulsory psychiatric care changed after the reform for patients in treatment both before and after the legal change (recurrent patients), and
- to analyse if those on CTOs in general and forensic psychiatry were the same patients as those on long leaves from compulsory psychiatric care before the change of the law.

## Methods

### Legal prerequisites

In Sweden, civil commitments are regulated by the Compulsory Psychiatric Care Act, according to which coercive care may be given only if the patient suffers from a serious mental disturbance, due to his or her mental state and general personal circumstances has an absolute need of inpatient psychiatric care, and objects to such care. The Forensic Psychiatric Care Act regulates care of patients sentenced to psychiatric care after committing a crime. The legal system claims all citizens to be responsible for their acts. However, if a forensic psychiatric evaluation establishes that a convicted criminal offender was suffering from a serious mental disorder at the time of the offence, the court has to sentence him or her to forensic psychiatric treatment in a secure hospital, not to imprisonment. Both laws permit decisions to let the patients be on temporary leave from hospital for limited periods of time, if it is in accordance with the treatment plan of the patient. When on leave, the patient is still legally regarded as an inpatient. By amendments of these Acts from the 1^st^ of September 2008, psychiatric outpatient compulsory care (OCC) and psychiatric outpatient forensic care (OFC) respectively were implemented. In both cases, compulsory psychiatric care has to start with an involuntary admission to psychiatric inpatient care. When the patient is assessed to be in need for further compulsory care, but not to be in need for inpatient care, the chief psychiatrist may apply to an administrative court for the patient to be transferred to CTO. When approving the application, the court may decide certain conditions that the patient has to comply with, for example to take medication, undergo other treatment, keep in touch with a certain person or institution, or not use intoxicants.

### Setting

Health care in Sweden is mainly run by Counties or Regions. The study was a register study conducted in Örebro County, in the southern central part of Sweden, with about 280 000 inhabitants. The psychiatric services of the county had in 2010 barely 900 employees, 136 beds and about 1 600 inpatients with circa 3 100 admissions and close to 46 000 days of inpatient treatment. More than 11 200 people had in the same year about 120 000 psychiatric outpatient visits [18].

### Material

Data were collected from the computerized patient administrative system (Infomedix) of the County Council from two periods of time: two years before (September 1, 2006 – August 31, 2008) and two years after the legal change (September 1, 2008 – August 31, 2010). These periods of two years each are in the following named period I and period II respectively. For each patient who had been involuntarily admitted once or more during one or both of these time periods, and for each treatment episode, the following information was gathered:

- legal status (compulsory psychiatric inpatient care, forensic psychiatric inpatient care, OCC, OFC)
- dates of admission, discharge, start and cessation of CTOs (period II only), and start and cessation of leaves
- sex and ICD-10 diagnoses.

Treatment episodes that were ongoing when the legal change came into force (1^st^ of September, 2008) were recorded as two separate episodes, one in period I (ending 31^st^ of August) and one in period II (starting 1^st^ of September). Ongoing episodes at the beginning (1^st^ of September 2006) and at the end (31^st^ of August, 2010) of the total data collection period were included.

The Swedish civic registration number was used to connect unique individuals to continuous treatment episodes comprising different forms of legal status and to identify individuals treated during both time periods. Patients on leave from compulsory psychiatric care are according to the County Council’s administrative procedures still registered as inpatients, and thus days on leave are included when days of inpatient treatment are counted. However, days actually spent in hospital may be regarded as more adequate information and are thus presented separately. Data were manually thoroughly checked and corrected to ensure data quality.

### Statistical analyses

The samples of patients before and after the legal change are partly independent and partly related, and the results of the descriptive analyses were not tested for statistical significance. For changes in the amounts of compulsory psychiatric care the Binomial test was used. Due to skew distributions, for recurrent patients changes in use of compulsory care were tested by the Wilcoxon signed-rank test and correlations between days on leave and CTOs by Spearman’s rho.

### Ethical considerations

Included patients were not informed about the study. To contact each individual and ask for consent to use administrative data regarding his or her involuntary psychiatric care, that took place a long time ago, was regarded as ethically problematic since reminders of past negative events may cause distress. It would also be practically difficult and probably result in a large number of dropouts. The study was approved by the Regional Research Ethics Board in Uppsala, Sweden (reference number 2012/310).

## Results

The number of involuntarily admitted patients was 524 in period I and 514 in period II. Of these, 62 and 67 respectively were forensic patients. The numbers of patients with leaves were 259 in period I and 223 in period II. In period II, 62 patients had CTOs (29 on OCC and 33 on OFC). The distributions on sex and diagnostic groups are shown in Table 1. Of all involuntarily admitted patients in both periods, around 50% were female. Around 35% had schizophrenia diagnoses and 20% mood disorders.

**Table 1:** **Distribution on sex and diagnoses (%)**

	All involuntarily admitted inpatients		Thereof, patients on leave		Period II, patients on CTOs ^a^	
	Period I ^b^	Period II ^c^	Period I ^b^	Period II ^c^	OCC ^d^	OFC ^e^
	n = 524	n = 514	n = 259	n = 223	n = 29	n = 33
Women	51	47	54	52	52	27
Diagnoses^f^	n = 637	n = 648	n = 423	n = 341	n = 48	n = 63
Psychoactive substance use (F10-19)	12,9	13,3	8,7	6,7	8,3	6,3
Schizophrenia etc. (F20-29)	34,1	37,3	39,5	43,7	68,8	34,9
Mood disorders (F30-39)	20,3	17,3	19,4	18,5	4,2	3,2
Anxiety etc. (F40-48)	5,8	4,2	4,3	3,2	-	-
Behavioral syndromes etc. (F60-69)	12,7	11,9	13,7	10,3	8,3	19,0
Intellectual disabilities (F70-79)	3,6	3,7	3,5	5,0	-	6,3
Developmental disorders (F80-89)	5,0	5,6	5,9	6,2	6,3	15,9
Behavioral and emotional disorders (F90-98)	2,7	3,4	3,3	4,4	4,2	14,3

^a^Community treatment orders ^b^The two year-period before the reform ^c^The two year-period after the reform ^d^Outpatient compulsory care ^e^Outpatient forensic care ^f^Some patients had more than one diagnosis.

The total number of leaves decreased from 2156 to 1750 (- 19%). The median and maximum as well as the average length of leaves decreased. Both the number of OCC decisions per patients (71/29) and OFC decisions per patient (79/33) were 2,4. On group level, OFC episodes were considerably longer than OCC episodes (Table 2).

**Table 2:** **Leaves from involuntary psychiatric inpatient care and community treatment orders (CTOs)**

	Leaves		CTOs (period II ^b^ )		
	Period I ^a^	Period II ^b^	OCC ^c^	OFC ^d^	Total
Patients	259	223	29	33	62
Decisions	2156	1750	71	79	150
Days	23 625	9 546	8 026	12 273	20 299
Md	18	16	182	343	249,5
Min	0	1	66	27	27
Max	730	344	707	686	707
Days/decision	11,0	5,5	113,0	155,4	135,3

^a^The two year-period before the reform ^b^The two year-period after the reform ^c^Outpatient compulsory care ^d^Outpatient forensic care.

The number of compulsory inpatient treatment episodes per patient was 1,4 in both periods (739/524 and 720/514 respectively). There was a 29% decrease from period I to period II in total inpatient days of compulsory psychiatric care, while days on leave decreased with 60%. Consequently, the days actually spent in hospital decreased less (nine percent). In period II there were more than 20 000 days of CTOs, and when comparing the sum of days at hospital and days on leave in period I with the sum of days at hospital, days on leave, and days of CTOs in period II there was a five percent increase from around 59 000 to 62 000 (Table 3).

**Table 3:** **Involuntary psychiatric care before and after the introduction of community treatment orders (CTOs)**

	Period I ^a^	Period II ^b ^	Change	p-value
Total number of patients	524	514	- 2%	0,780
Inpatient treatment episodes	739	720	- 3%	0,637
Inpatient days	59 423	42 060	- 29%	<0,001
Thereof, number of days on leave	23 625	9 546	- 60%	<0,001
Days in hospital	35 798	32 514	- 9%	<0,001
Days of CTOs	-	20 299		
Days on leave + CTOs	23 625	29 845	+ 26%	<0,001
Days in hospital + on leave + CTOs	59 423	62 359	+ 5%	<0,001

^a^The two year-period before the reform ^b^The two year-period after the reform.

### Recurrent patients

Of all patients in the study, 173 were involuntarily admitted during both period I and period II (recurrent patients). They had around 300 inpatient treatment episodes each period. Their inpatient days and days on leave decreased from period I to period II, and there was a tendency of increased number of days on leave plus on CTOs in period II compared to the number of days on leave in period I (p = 0,095). In all, there was no significant change in total days at hospital, on leave, and/or on CTOs (Table 4).

**Figure 1:**
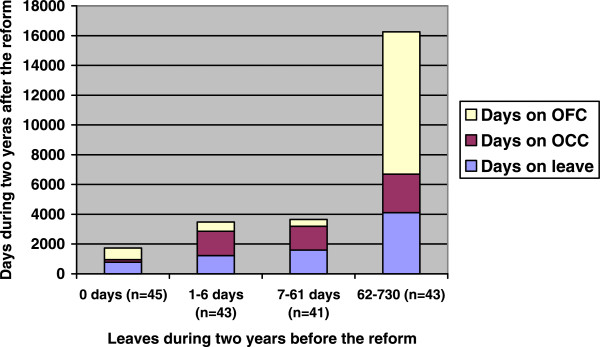
**Recurrent patients (n = 172) – days on leave during two years before the reform versus days on leave, outpatient compulsory care (OCC) and outpatient forensic care (OFC) during two years after the reform.**

All recurrent patients, except one with missing data, were categorized into four groups based on the quartiles of length of leaves in period I. Those 25% of patients with the longest leaves (62–730 days) in period I had more days on leave and on CTOs in period II than the other recurrent patients (Figure 1). For involuntarily admitted patients in general psychiatry (n = 118), days on leave in period I were significantly correlated with days on leave (0,446; p < 0,001) and days on OCC (0,337; p < 0,001) in period II. For forensic patients (n = 48), days on leave in period I were not associated to days on leave (0,131; p = 0,375) but to days on OFC in period II (0,523; p < 0,001). Six patients, who were involuntarily treated both in general and in forensic psychiatry, were excluded from the correlation analyses.

**Table 4:** **Recurrent involuntarily admitted patients (n = 173) before and after the introduction of community treatment orders (CTOs)**

	Period I ^a^	Period II ^b^	Change/p
Inpatient occasions	309	295	-5%
Md (25^th^; 75^th^ percentile)	1 (1; 2)	1 (1; 2)	0,717
Inpatient days	43 967	28 914	- 34%
Md (25^th^; 75^th^ percentile)	91 (30; 525)	73 (22; 202)	<0,001
Thereof, number of days on leave	17 515	7 707	-56%
Md (25^th^; 75^th^ percentile)	10 (0; 114)	4 (0; 65)	<0,001
Days in hospital	26 452	21 207	- 20%
Md (25^th^; 75^th^ percentile)	48 (20; 172)	30 (8; 140)	0,010
Days of CTOs	-	17 404	
Md (25^th^; 75^th^ percentile)		0 (0; 116)	
Days on leave + CTOs	17 515	25 111	+ 43%
Md (25^th^; 75^th^ percentile)	10 (0; 114)	15 (0; 239)	0,095
Days in hospital + on leave + CTOs	43 967	46 318	+ 5%
Md (25^th^; 75^th^ percentile)	91 (30; 525)	118 (22; 582)	0,573

^a^The two year-period before the reform ^b^The two year-period after the reform.

## Discussion

In a review of the effect of CTOs on service use [19] the authors conclude that there now is robust evidence that CTOs have no significant effect on service use outcomes, and that non-randomised studies report conflicting results. Like the studies referred to in the introduction [7, 8], the present study indicates that the use of CTOs may have some positive effect on hospitalization rates. In a Spanish study of 91 patients under involuntary out-patient commitment [20] emergency room visits, admissions, and lengths of hospitalisations were reduced in a two-year period following the initiation of this form of commitment. In our study based on data from one Swedish county, the main findings were that the total number of days of psychiatric in-patient care actually spent in hospital was reduced with nine percent after the implementation of CTOs, and that the recurrent patients had 20% less days in hospital in the two-year period after the reform than before. However, the number of days on leave plus days under CTOs increased with 26% among all patients and with 43% among recurrent patients. The number of days under any form of compulsory care (in hospital, on leave, and under CTOs) for all involuntarily admitted patients increased with five percent. This indicates that legal sanctioning of an additional form of compulsory care may lead to an increase in the total use of coercion in psychiatry. Obviously, the implementation of CTOs in Sweden, at least in Örebro County, was followed by a marked increase in the use of the most controversial form of involuntary treatment, namely the use of coercive psychiatric care in the community.

The use of temporary leaves from involuntary psychiatric care was strongly reduced after the implementation of CTOs, and those patients with long periods of leaves before also were those with most CTOs after the reform. This was true for patients both in general and in forensic psychiatry. These results indicate that one of the main aims of this reform in Sweden, to replace long leaves from compulsory psychiatric inpatient care with CTOs, was fulfilled. One may argue that this is obviously an expected finding, but unintended effects of law reforms have been reported in the literature [21]. Our findings support the conclusion of an evaluation of the NBHW based on examinations of administrative court cases [16], that the reform has reached the intended patients. Still, in our study patients with many days of CTOs also had more days on leave in period II than other patients, indicating that some patients are more subjected to both forms of compulsory care in the community than others.

CTOs were in our study used on relatively far more patients in forensic psychiatric care than in general psychiatry. This is in line with another evaluation of the reform by the NBHW [17], which showed that the Swedish legal regulations of CTOs worked better in forensic than in general psychiatry. CTOs require a working collaboration with local social services, and the forensic psychiatric services have probably been more successful in establishing such collaboration than psychiatry in general.

A strength of our study is that it comprises all involuntarily treated psychiatric patients in a defined catchment area during four years. The data were as far as possible checked for completeness and correctness. As ongoing treatment episodes at the start and at the end of the four-year period were included, the median length of treatment episodes is somewhat underestimated. Ongoing treatment episodes when CTOs came into force were divided into two and thus the number of episodes is slightly overestimated. Anyway data from both period I and period II are calculated in the same manner and thus comparable.

The main limitation is that the study includes one county, only. Published data indicate that the use of OCTs varies considerably within and between different countries [1, 5, 13, 22], and it is not obvious that our results can be generalized to Sweden as a whole or to other countries. More studies of both intended and unintended effects of CTOs are called for. Current evidence clearly does not provide enough support for CTOs. However, the justification of CTOs is an ethical question which cannot solely be decided by evidence [3, 4]. With regard to the present study, a basic ethical question is if restrictions of individual liberty in the community may be justified by decreased time involuntarily spent in hospital. The present study gives no answer to this or to questions about, for example, the impact of CTOs on quality of life for patients and their families. Empirical data is however needed to provide support for health policy decisions, not least when it comes to the extension of coercive power of psychiatry into the community.

## Conclusions

Our study suggests that one of the main intentions of the legislator with introducing CTOs in Sweden, namely to replace long leaves from involuntary psychiatric inpatient care, has been fulfilled at least in the short run. Patients with the longest leaves before the reform had more days on CTOs after the reform than other recurrent patients. The total number of involuntary days spent in hospital decreased, but at the same time the use of coercive psychiatric care outside hospital, i.e. leaves and CTOs, increased. There was also a small increase in the use of in- and outpatient coercive psychiatric care as a whole. Adding an additional legal coercive instrument in psychiatry may increase the total use of coercion.

## Abbreviations

CTOs: Community treatment orders
NBHW: National Board of Health and Welfare
OCC: Outpatient compulsory care
OFC: Outpatient forensic care.
